# Paternal B Vitamin Intake Is a Determinant of Growth, Hepatic Lipid Metabolism and Intestinal Tumor Volume in Female Apc^1638N^ Mouse Offspring

**DOI:** 10.1371/journal.pone.0151579

**Published:** 2016-03-11

**Authors:** Julia A. Sabet, Lara K. Park, Lakshmanan K. Iyer, Albert K. Tai, Gar Yee Koh, Anna C. Pfalzer, Laurence D. Parnell, Joel B. Mason, Zhenhua Liu, Alexander J. Byun, Jimmy W. Crott

**Affiliations:** 1 Jean Mayer USDA Human Nutrition Research Center on Aging at Tufts University, Boston, Massachusetts, United States of America; 2 Friedman School of Nutrition Science and Policy at Tufts University, Boston, Massachusetts, United States of America; 3 Tufts Center for Neuroscience Research, Neuroscience Department, Tufts University School of Medicine, Boston, Massachusetts, United States of America; 4 Molecular Cardiology Research Institute, Tufts Medical Center, Boston, Massachusetts, United States of America; 5 Tufts University Core Facility, Tufts University School of Medicine, Boston, Massachusetts, United States of America; 6 School of Public Health and Health Sciences, UMass Amherst, Amherst, Massachusetts, United States of America; National Cancer Institute, UNITED STATES

## Abstract

**Background:**

The importance of maternal nutrition to offspring health and risk of disease is well established. Emerging evidence suggests paternal diet may affect offspring health as well.

**Objective:**

In the current study we sought to determine whether modulating pre-conception paternal B vitamin intake alters intestinal tumor formation in offspring. Additionally, we sought to identify potential mechanisms for the observed weight differential among offspring by profiling hepatic gene expression and lipid content.

**Methods:**

Male Apc^1638N^ mice (prone to intestinal tumor formation) were fed diets containing replete (control, CTRL), mildly deficient (DEF), or supplemental (SUPP) quantities of vitamins B_2_, B_6_, B_12_, and folate for 8 weeks before mating with control-fed wild type females. Wild type offspring were euthanized at weaning and hepatic gene expression profiled. Apc^1638N^ offspring were fed a replete diet and euthanized at 28 weeks of age to assess tumor burden.

**Results:**

No differences in intestinal tumor incidence or burden were found between male Apc^1638N^ offspring of different paternal diet groups. Although in female Apc^1638N^ offspring there were no differences in tumor incidence or multiplicity, a stepwise increase in tumor volume with increasing paternal B vitamin intake was observed. Interestingly, female offspring of SUPP and DEF fathers had a significantly lower body weight than those of CTRL fed fathers. Moreover, hepatic trigylcerides and cholesterol were elevated 3-fold in adult female offspring of SUPP fathers. Weanling offspring of the same fathers displayed altered expression of several key lipid-metabolism genes. Hundreds of differentially methylated regions were identified in the paternal sperm in response to DEF and SUPP diets. Aside from a few genes including Igf2, there was a striking lack of overlap between these genes differentially methylated in sperm and differentially expressed in offspring.

**Conclusions:**

In this animal model, modulation of paternal B vitamin intake prior to mating alters offspring weight gain, lipid metabolism and tumor growth in a sex-specific fashion. These results highlight the need to better define how paternal nutrition affects the health of offspring.

## Introduction

Evidence is rapidly accruing that underscores the importance of maternal diet in determining the risk of various health outcomes in offspring, including cardiovascular disease [1], obesity [2], diabetes [2], psychological disorders [3, 4], and certain cancers [5]. Using a mouse model of colorectal cancer, we have shown that maternal depletion of vitamins B_6_, B_12_, riboflavin and folate increases the risk of invasive intestinal cancer in offspring, whereas supplementation reduces the overall incidence of intestinal tumors [6], and similar results were reported in a different mouse model of CRC [7].

In addition to the well accepted importance of maternal diet, emerging evidence suggests that paternal diet prior to conception also has the potential to influence offspring health. For example, high fat feeding to male rats prior to mating induced abnormal glucose tolerance and insulin secretion in their female offspring that was associated with a depletion of large pancreatic islets and a multitude of gene expression changes [8]. Others have shown that feeding male mice low protein diets prior to mating caused reductions in cholesterol esters, phosphatidylethanolamine and free cholesterol in offspring blood. Furthermore, Pparα, a major regulator of hepatic lipid metabolism, was hyper-methylated and down-regulated in the liver of offspring from low protein fathers [9]. In addition to paternal macronutrients, micronutrient intake also appears to be important. For example, paternal folate deficiency reduced body weight, length, hepatic folate and brain *Igf2* expression in fetal offspring [10]. Others have shown that severe folate deficiency in future sires, beginning *in utero* and continuing to the time of mating, caused a significant elevation of craniofacial and musculoskeletal malformations in offspring [11].

One potential mechanism by which maternal and paternal diet may influence offspring health is by modulating epigenetic patterns in germ cells or the developing embryo. Much of the mammalian genome is actively demethylated shortly after fertilization [12], however the methylation state of certain loci may be retained across generations. The best known examples of this are imprinted genes [13], in which one parental allele is epigenetically silenced, and metastable epialleles, which are alleles that can be differentially expressed in genetically identical individuals due to differences in epigenetic modifications such as methylation [14]. Importantly, it is becoming clear that the methylation of specific imprinted genes and metastable epialleles is sensitive to parental nutritional status. For example, the maternally-imprinted *IGF2* gene is reported to be relatively hypermethylated in the offspring of mothers taking peri-conceptional folate supplements [15, 16] and relatively hypomethylated in the offspring of obese fathers [17]. The sensitivity of metastable epiallele methylation to maternal nutritional status was first demonstrated in Agouti viable yellow [18–20] and Axin fused [21] mice and later in humans [22, 23].

Based on our observations that maternal B vitamin supplementation can suppress tumor formation in offspring and also accumulating evidence that altered paternal intake of macro and micronutrients can influence several phenotypes in the offspring, we sought to determine whether modulating paternal B vitamin intake affects offspring tumorigenesis in the Apc^1638N^ mouse model of CRC. Contrary to our expectations, we observed no differences in tumor incidence between offspring of different paternal diet groups but did note a significant step-wise increase in the volume of tumors with increasing paternal B vitamin intake in female but not male offspring. Interestingly paternal B vitamin intake affected offspring body weight in female offspring. To better understand the effect of paternal B vitamin on offspring physiology in general we profiled hepatic gene expression patterns. An enrichment of lipid metabolizing genes in female offspring of supplemented fathers was coupled with a marked elevation in hepatic triglycerides. Our observations support the concept that alterations in paternal B vitamin intake can have diverse impacts on offspring physiology that persist through adulthood and highlight the need to better characterize how paternal diet might program health and disease risk in offspring.

## Materials and Methods

### Animal study

All animal procedures were approved by the Institutional Animal Care and Use Committee (IACUC) of the Jean Mayer USDA Human Nutrition Research Center on Aging at Tufts University. For the duration of the experiment, all animals were housed in a 12 h light–dark cycle at 23°C and provided with free access to water.

The Apc^1638N^ mouse model of human intestinal cancer was utilized. Mice possessing one allele with this mutation in the *Apc* gene (amino acid 1638) spontaneously develop small intestinal neoplasms beginning at approximately 10 weeks of age, some of which remain as adenomas and some which advance to adenocarcinomas over the ensuing 6–8 weeks [24]. Although the predilection for developing small, rather than large, intestinal tumors is a common phenomenon in genetically-engineered models of CRC—such as the widely utilized Apc^min^ mouse—the small intestinal tumorigenesis in the Apc^1638N^ mouse is an appropriate tool for modelling CRC because its latency, burden and response to dietary perturbations such as inadequacy of 1-carbon nutrients and obesity [25, 26] closely match the situation in the human colon.

**Fig 1:**
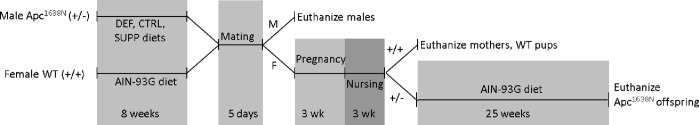
Design of animal experiment. Apc1638N (+/−), mice heterozygous for truncation mutation in the *Apc* gene; WT (+/+), C57BL6/J mice wild-type for Apc gene; DEF, B-vitamin deficient; CTRL, B-vitamin replete; SUPP, B-vitamin supplemented; M, male; F, female.

Weanling (21 day old) male F_0_ Apc^1638N^ mice were group pair-fed amino acid-defined diets with deficient (DEF), control (CTRL), or supplemental (SUPP) quantities of vitamins B_6_, B_12_, riboflavin, and folate (See Table 1. Dyets Inc., Bethlehem, PA) for 8 weeks prior to mating. As described in our prior publication [27], these diets were designed expressly to induce a very mild degree of deficiency that only diminishes tissue levels of the nutrients by 50–70%, as opposed to the moderate-to-severe deficiencies utilized in most rodent studies that often decrease tissue levels by ten-fold or more. Weanling wild type female C57BL/6J mice (one for every male) were maintained on replete AIN-93G diet *ad libitum* for approximately 8 weeks before mating. The study design is shown in Fig 1.

**Table 1:** B vitamin composition of parental and offspring diets.

	Diet	Folate (mg/kg)	Vit. B2 (mg/kg)	Vit. B_6_ (mg/kg)	Vit. B_12_ (μg/kg)
Apc^1638N^ fathers*	DEF	0.5	2.0	2.0	10.0
	CTRL	2.0	6.0	7.0	50.0
	SUPP	8.0	24.0	28.0	200.0
Mothers & offspring †	AIN-93G	2.0	6.0	7.0	25.0

* Fathers were pair-fed amino-acid defined diets with differing B vitamin contents starting 8 weeks before mating, until the end of mating.

† AIN-93G diet was provided to mothers *ad libitum* beginning approximately 8 weeks before mating and lasting through pregnancy and nursing of offspring. Offspring were fed the diet *ad libitum* until the time of sacrifice (either at weaning or 28 weeks of age). DEF, B vitamin deficient; CTRL, B vitamin replete; SUPP, B vitamin supplemented

After 8 weeks on diets, pairs of female and male mice were mated for five consecutive nights. In order to prevent possible confounding by males consuming the female’s diet (or vice versa), food was withheld during overnight mating periods. During the day mating pairs were separated to their respective cages and fed assigned diets. After the completion of mating, at approximately 12 weeks of age, F_0_ males were euthanized by CO_2_ asphyxiation, cervical dislocation and exsanguination by cardiac puncture. Testes were removed and sperm cells were isolated from the caudal epididymis according to the method of Liu et al [28]. Sperm motility was estimated by averaging the percentage of sperm that were moving in two (1 mm^2^) squares on opposite corners of a hemocytometer. An average of 189 ± 15 (SEM) sperm cells were counted per sample.

During gestation and nursing, dams continued to be fed AIN-93G (*ad libitum*). Approximately 21 days after birth, F_1_ pups were weaned and dams euthanized by CO_2_ asphyxiation and cervical dislocation. Litter size was not standardized. Weanling offspring were genotyped from tail snips: Apc^1638N^ heterozygous offspring were maintained on replete AIN-93G diet (*ad libitum)* until 28 weeks of age in order to assess tumor formation (homozygous mutants are embryonic lethal). Because wild type offspring do not develop tumors they were euthanized after weaning. Upon sacrifice, blood (for plasma) and liver were collected for both genotypes and stored at -80°C.

For Apc^1638N^ offspring, the small intestine was removed, flushed through with cold PBS, opened longitudinally and rinsed again in PBS with a cocktail of protease inhibitors (Roche, Indianapolis, IN). The SI was then inspected under a dissecting microscope for the presence of tumors by a blinded observer. Two dimensions of each tumor were measured with digital calipers (in mm) and the third dimension (D) imputed as the average of these. Volume was calculated as D1 x D2 x D3. Tumors were then excised and fixed in formalin for later embedding, sectioning and slide preparation. Tumors were classified as adenomas or invasive carcinomas from H&E-stained tumor slides by a blinded, expert rodent pathologist.

### Paternal blood analyses

In paternal plasma, vitamin B_6_ (as pyridoxal phosphate) concentrations were measured following protein precipitation using a radioenzymatic assay [29], while vitamin B_12_ and folate concentrations were measured by chemiluminescent immunoassay using the Immulite 1000 (Siemens, Tarrytown, NY). Riboflavin status was assessed in packed red blood cells by the glutathione reductase activation coefficient [30]. Plasma glucose (CrystalChem, Downers Grove, IL) and insulin were measured by colorimetric (EMD Millipore, Billerica, MA) and ELISA assays respectively, according to manufacturer’s protocols.

### Sperm DNA methylation

Genome-wide DNA methylation patterns in the sperm of 24 fathers (n = 8/gp) were measured using Methylated DNA Immunoprecipitation (MeDIP)-chip [31]. First, sperm cells were digested and DNA was extracted using phenol/chloroform. Sample quality was confirmed by a 260/280 ratio >1.8, as measured using a Nanodrop Spectrophotometer ND-1000 (Thermo Scientific, Wilmington, DE). DNA was then sheared to 200-1000bp in length by sonication (Sonifier Cell Disruptor 350.VWR, Radnor, PA) with confirmation by agarose gel electrophoresis. MeDIP was performed using the MagMeDIP kit (Diagenode, Denville, NJ), which utilizes magnetic beads to yield one methyl-enriched and one non-immunoprecipitated (input) sample per animal. Beads were washed and DNA was eluted and purified using the IPure kit (Diagenode, Denville, NJ). Immunoprecipitation quality was confirmed by qPCR of methylated Tsh2β, unmethylated Gapdh and internal methylated controls. Whole genome amplification was done by PCR (WGA2Sigma, St Loius, MO), followed by purification using the QIAquick PCR Purification Kit (Qiagen, Valencia, CA). Methyl-enriched DNA and the non-immunoprecipitated control (input) DNA from each animal were labeled using Cy5 and Cy3 fluorophores, respectively, using a Klenow polymerized amplification reaction. Fiducial controls were then added to the samples in order to align arrays during analysis. As there were 3 arrays per slide, the labeled samples were re-suspended in unique Sample Tracking Controls (STC) to preclude cross-contamination. Sample pairs were then dissolved in hybridization buffer and co-hybridized to a NimbleGen 3x720K RefSeq plus CpG Island DNA Methylation array using the NimbleGen HS4 Hybridization System (Roche NimbleGen, Madison, WI). Arrays covered all known gene promoters (20,404), transcripts (22,881) and CpG islands (15,980) in the mouse genome. The slides were then washed to eliminate non-specific binding and immediately dried using the NimbleGen accelerated Microarray Dryer (Roche NimbleGen, Madison, WI). They were then scanned with 532m and 635m lasers using the MS 200 Microarray Scanner (Roche NimbleGen, Madison, WI) at 2μm resolution.

Differentially methylated regions (DMRs) between the diet groups were identified using the Bioconductor/R software package, CHARM (comprehensive high-throughput arrays for relative methylation) [32]. Because CHARM was originally developed to analyze the ratio of DNA enriched in unmethylated DNA to input DNA, we modified the code to be applicable to our MeDIP enrichment method by inverting the M ratio to MeDIP/input and eliminating the percent methylation calculation. Genomic regions were considered to be differentially methylated between groups if a minimum of 3 adjacent probes had p values <0.005. DMRs were annotated to the nearest gene using GREAT (Genomic Regions Enrichment of Annotations Tool) [33].

### Offspring gene expression profiling

To investigate the potential impact of paternal diet on offspring physiology we profiled genome-wide hepatic gene expression in weanling wild type offspring by RNA sequencing. We chose to do this in weanling, as opposed to adult offspring, because we reasoned that changes induced by paternal diet might attenuate over time. RNA was isolated from the livers of 30 offspring (n = 5 males, 5 females per paternal diet group) using the Ambion® RiboPure™ Kit (Life Technologies, Grand Island, NY). RNA quality was assessed using the 2100 Bioanalyzer system (Agilent Technologies, Santa Clara, CA) to verify that the RNA Integrity Number (RIN) was greater than 8. Library preparation was done using the TruSeq RNA Sample Preparation Kit v2 (Illumina, San Diego, CA), with 1 μg of input RNA per sample. Quality was assessed using the Fragment Analyzer (Advanced Analytical, Ames, IA). Single-end sequencing was performed on the HiSeq 2500 (Illumina, San Diego, CA). The read length was 51 bases. The demultiplexed FASTQ files were generated using CASAVA 1.8.2 (Illumina), and the QC reports were generated with FastQC. Samples were considered of acceptable quality if the mean quality score was at least 30 and the percentage of bases with a quality score of ≥ 30 was at least 85%. An average of 17,942,889 ± 307,516 (SEM) reads were generated per sample.

RNA-Seq reads from the FASTQ files were aligned to the mouse genome (mm10, GRCm38) using TopHat v2.0.11 [34] and then processed with Cufflinks v2.2.1 [35] to assemble transcripts. Mean fragment length (230.37 bp), standard deviation of the distribution of fragment lengths (6.12bp),—*frag-bias-correct*, and—*multi-read-correct* were provided as options, and a reference GTF file (mm10) was provided with the–G option in order to estimate known isoform expression. The Cufflinks assemblies were merged together using Cuffmerge with the reference GTF and reference genome files provided. Cuffdiff was used to identify differentially expressed genes, both with males and females together and separately. Mean fragment length, standard deviation of the distribution of fragment lengths,—*max-bundle-frags 10000000*,—*frag-bias-correct*, and—*multi-read-correct* were included as options. For quality control, a summary of average FPKM (Fragments Per Kilobase of transcript per Million mapped reads) values per sample was generated in the R to confirm that there were no apparent outliers in terms of mean, median, minimum, and maximum values. Several common housekeeping genes were also plotted to check for outliers (Actb, Ppia, Hmbs, Pgk1, Hprt, Sdha, Tbp, Rpl13a, B2m, Gusb, Tubb4a, Ywhaz, Alas1, Gapdh) and no samples appeared abnormal by this measure. Genes with a transcript length of less than the average fragment length (230bp) were removed using R, because Cufflinks is known to overestimate the expression values of short transcripts. Significance of differential expression was accepted when q<0.05 (default Cuffdiff output).

Ingenuity Pathway Analysis (IPA®; Qiagen, Redwood City, CA) was used to identify functional categories enriched within our differentially expressed genes and to overlay observed changes upon networks empirically determined to interact. The IPA software calculates the p values for the functional analyses using the right-tailed Fisher’s Exact Test. The network score is a negative log p value of the Fisher exact test, which is testing whether these genes are grouped by chance. A network score of 2 means a 1/100 chance that this grouping would be observed by chance.

### Offspring DNA methylation analyses

Genomic DNA was isolated from offspring liver using the ‘DNeasy Blood and tissue kit’ (Qiagen, Valencia, CA) and 500 ng was bisulfite converted using the ‘EZ DNA Methylation-lightning’ kit (Zymo, Irvine, CA). CpG islands immediately upstream or surrounding the transcription start site of *Elovl6*, *Acaca* and *Gpam* were identified using MethPrimer Software [36]. For Igf2, a region lying between Igf2 and the downstream H19 gene known as the H19 differentially-methylated region [37] was targeted. PCR primers and sequencing primers were designed using PyroMark Assay Design software (v 2.0.2.15)(See S1 Table). Amplicons for sequencing were generated using the PyroMark PCR kit and sequencing was performed on a Pyromark Q24 instrument using the PyroMark Q24 Advanced CpG kit (n = 8 and 4/gene for females and males respectively). Sequencing runs were setup and data analyzed with PyroMark Q24 Advanced software (v3.0.0). All sequencing reagents and software were from Qiagen (Valencia, CA).

### Offspring blood and liver analytes

To determine whether differential B-vitamin intake by fathers affects offspring vitamin status, hepatic folate content was assessed with the *Lactobacillus casei* microbiological assay preceded by conjugase treatment [38] in weanling wild type (n = 8/gp) and 7 month old Apc^1638N^ F_1_ pups (n = 32-37/gp). Because body weight differences were observed among Apc^1638N^ offspring of different paternal diet groups, we measured plasma insulin and leptin from blood collected at the time of sacrifice in order to determine whether body weight differences were due to differences in metabolic programming and body fat. Plasma insulin (CrystalChem, Downers Grove, IL) and leptin (EMD Millipore, Billerica, MA) were measured by ELISA according to manufacturers’ instructions (n = 9-13/gp). Because *‘lipid metabolism’* was one of the top categories from the IPA analysis of differentially expressed genes we also measured hepatic lipid content in adult Apc^1638N^ offspring. Briefly, lipids were extracted by homogenizing ~100mg of liver in chloroform:methanol (2:1) and treating with H_2_SO_4_ [39]. Total cholesterol and triglycerides were measured using colorimetric assays on the AU400 instrument (Beckman Coulter, Indianapolis, IN) according to the manufacturer’s protocols (n = 13/gp). De-lipidated liver tissue was digested in 1N NaOH and the BCA assay (Pierce, Rockford, IL) was used to measure protein content. Hepatic TG was also assessed histologically in female offspring in a randomly selected potion of the liver. Briefly, sections were prepared from frozen tissue (n = 5/gp), stained with Oil Red-O, and photographed under 100x magnification (Olympus CX2 microscope, DP72 camera and DP2-BSW v2.2 software. Olympus, Center Valley, PA). The percentage area stained by Oil Red-O was calculated by thresholding in ImageJ [40] and values obtained from 3 images were averaged per sample. The abundance of inflammatory cytokines (Tnf, Il1b, Ifn, Il6) in the liver was measured by electrochemiluminescence array and Sector S600 imager according to manufacturer’s protocols (Mesoscale Discovery, Rockville, MD. n = 6-7/gp).

### Statistical analyses

All data are reported as mean ± SEM. Between-group comparisons of various endpoints were made with single or two-factor ANOVA and associations between variables tested using Pearson correlation. Analyses of fathers’ and Apc^1638N^ offspring body weight over time were performed using repeated measures ANOVA. Comparisons of reproductive success; genotype and sex distribution of offspring; and tumor incidence, grade, and location were done using Chi Square test. Calculations were performed in SAS v9.3 (SAS Institute, Cary, NC) and Systat v11 (San Jose, CA) and significance was accepted at P<0.05. Commonality between gene lists was assessed using the Venn diagram tool of Oliveros [41].

## Results

### Paternal blood vitamins and body weight

As intended, there was a stepwise increase in paternal plasma folate, vitamin B_6_ and vitamin B_12_ concentrations with increasing vitamin content of diet (Table 2). Riboflavin status, which was measured in a reciprocal fashion by the RBC glutathione reductase activation coefficient, was lower in the deplete group but equal in the CTRL and SUPP groups: such is to be expected with an activation coefficient assay because the apoenzyme becomes fully saturated with the riboflavin co-factor at dietary intakes exceeding the basal needs of the animal and therefore the assay does not detect supraphysiologic concentrations of the vitamin.

**Table 2:** Effect of differential B vitamin intake on blood B vitamin concentration in fathers.

	DEF	CTRL	SUPP	p value
Riboflavin (act. coeff.)**	1.25 ± 0.02*	1.18 ± 0.01	1.18 ± 0.01	0.001
Vitamin B_6_/PLP (nmol/L)	70.4 ± 8.5*	282.8 ± 20.5	663.8 ± 39.9*	<0.0001
Vitamin B_12_ (pmol/L)	1.2 ± 0.1*	10.3 ± 1.1	17.2 ± 0.2*	<0.0001
Folate (nmol/L)	36.7 ± 3.6*	118.5 ± 12.0	192.3 ± 16.53*	<0.0001

Values are mean ± SEM.

* Significantly different from CTRL (p<0.05).

** Riboflavin activity coefficient is reciprocal to actual concentrations. DEF, B vitamin deficient; CTRL, B vitamin replete; SUPP, B vitamin supplemented. N = 10, 11, 8, respectively.

Throughout the paternal diet intervention, fathers were weighed weekly. Mice receiving the DEF diet had modestly lower body weights than those receiving the CTRL diet from weeks 5–9 (S1 Fig): by week 9 of the diet intervention (mating week), the DEF groups was 10.1% lower than the CTRL (p<0.05). They nevertheless showed no outward signs of diminished activity or illness. By week 9, the SUPP group was also significantly lower in body weight than the CTRL group (5.4%; p<0.05. S1 Fig). Despite the observed differences in body weight, there were no significant differences in blood insulin and glucose concentrations between groups (p>0.05. S2 Table). As food was provided for only 8 hours each day (withheld during non-mating period) there was a slight but significant decrease in body weight from week 8 to week 9 (p<0.0001), but there was no significant interaction with diet (p = 0.6).

### DNA methylation in fathers sperm

Compared to fathers consuming the CTRL diet, there were 840 and 470 differentially methylated regions identified in the sperm DNA of DEF and SUPP fathers respectively (S3 and S4 Tables). These DMRs were mapped to 959 and 550 genes respectively. There are 114 genes that changed in both comparisons.

### Reproductive statistics

Paternal diet did not significantly affect the percentage of sperm that were motile, reproductive success or offspring sex distribution (S5 Table). No differences in Apc genotype distribution by diet were observed either on an individual father level, or in the offspring population as a whole (S5 Table. Chi-square p = 0.63). As homozygosity for the Apc^1638N^ mutation is embryonic lethal, this allele cannot be in Hardy-Weinberg equilibrium.

### Offspring physiology

**Fig 2:**
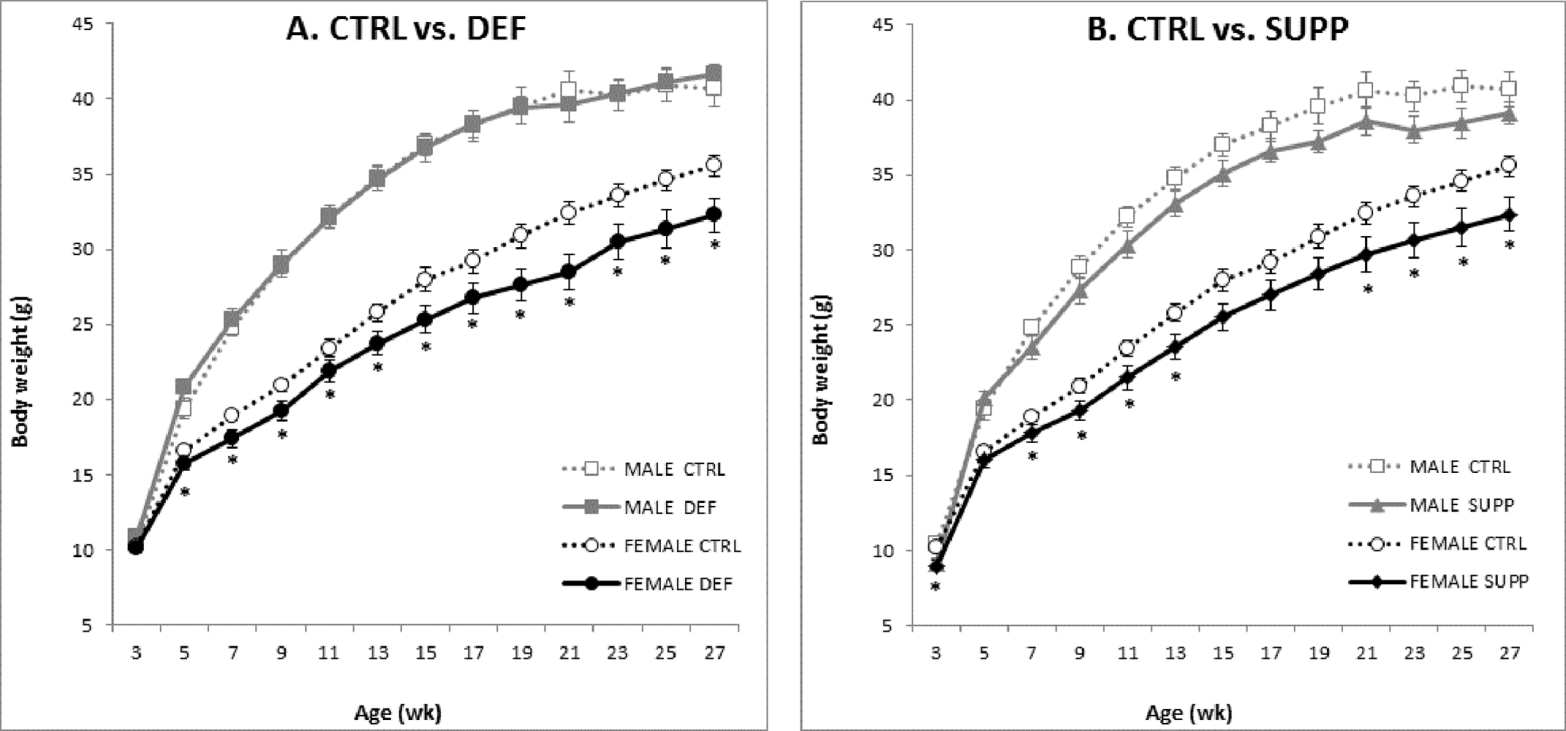
Body weight of Apc^1638N^ offspring of fathers fed diets differing in B vitamin content. **A)** Body weight of DEF compared to CTRL offspring, by sex. **B)** Body weight of SUPP compared to CTRL offspring, by sex. Repeated-measures ANOVA for paternal diet effect p = 0.64 and 0.02 in males and female offspring respectively. Paternal diets: DEF, B-vitamin deficient; CTRL, B-vitamin replete; SUPP, B-vitamin supplemented.* denotes significant difference (in females) compared to CTRL (p ≤0.05) by t-test. n = 14–22 per group.

No significant differences in hepatic folate content were observed between weanling wild type or adult Apc^1638N^ offspring of different paternal groups (Table 3). Weight at weaning was not affected by Apc genotype in males or female offspring of any group (Fig 2. p>0.05). Combining pups of both genotypes, paternal diet had no impact on weaning weight for male or female offspring (p>0.05). Considering the growth curve of the Apc mice retained for tumor studies between group differences in body weight became apparent; female offspring of DEF and SUPP fathers were significantly lighter than those of CTRL fathers over the entire 25 week study (Repeated measures ANOVA p<0.05). At the final time point the weight of DEF and SUPP offspring was 3.28g (9.2%) and 3.20g (9.0%) less than that of their CTRL counterparts, respectively (Fig 2). Interestingly, the body weight of male offspring was not affected significantly by paternal diet (p> 0.05), although there was a tendency over the entirety of the study for offspring of SUPP fathers to be lighter than those of CTRL fathers (Fig 2B). Despite the observed difference in body weight there was no significant differences in plasma leptin or insulin between adult offspring of different paternal diet groups (S6 Table).

**Table 3:** Hepatic folate content of weanling wild-type and adult Apc^1638N^ offspring of fathers consuming different quantities of B vitamins.

Offspring age (genotype)	Sex	DEF	CTRL	SUPP	p value
Weanling (wild-type)	Female	11.6 ± 0.7 (8)	12.4 ± 0.6 (8)	10.3 ± 0.7 (8)	0.10
	Male	10.5 ± 0.7 (8)	11.2 ± 0.6 (10)	11.9 ± 0.8 (8)	0.44
	All	11.1 ± 0.5 (16)	11.8 ± 0.4 (18)	11.1 ± 0.5 (16)	0.58
7 month-old (Apc^1638N^)	Female	12.1 ± 0.7 (15)	10.3 ± 0.4 (24)	10.9 ± 0.4 (22)	0.06
	Male	11.7 ± 0.7 (17)	12.2 ± 0.9 (14)	11.0 ± 1.0 (15)	0.61
	All	11.9 ± 0.5 (32)	11.0 ± 0.4 (38)	10.9 ± 0.5 (37)	0.31

Offspring liver folate content (μg/g liver tissue). Values are mean ± SEM. Paternal diets, DEF, B vitamin deficient; CTRL, B vitamin replete; SUPP, B vitamin supplemented. Sample size is in parentheses.

### Intestinal tumors in Apc^1638N^ offspring

No significant differences in tumor incidence (i.e. percent of mice that developed intestinal tumors) were found between offspring of different paternal diet groups. Small intestinal tumors were observed in 21 of 32 (65.6%), 22 of 36 (61.1%), and 23 of 39 (59%) offspring of DEF, CTRL and SUPP fathers, respectively (Chi-square p = 0.85. Table 4). Similarly, paternal diet group had no bearing on the mean number of tumors per mouse, or the percent of tumors that were invasive carcinomas. Further, when data from both sexes were combined, the total or average tumor volume in offspring did not differ between groups (Table 4). Offspring gender was a significant determinant of tumorigenesis; male mice had a higher tumor incidence (81.25 vs. 45.8%, Chi-square p = 0.0002), tumor multiplicity (1.28 vs. 0.54 tumors/mouse, p< 0.0001) and tumor volume (70.6 vs. 18.9 mm^3^/mouse, p = 0.001) than female mice. When considering male and female offspring separately there was still no effect of paternal diet on tumor incidence, multiplicity, or invasiveness. However, amongst females with tumors, there was a statistically significant stepwise increase in tumor volume with increasing paternal B vitamin intake (p_trend_ = 0.04). The magnitude of this effect was substantial: compared to DEF offspring, tumors in SUPP offspring had approximately 160% greater volume (p = 0.04).

**Table 4:** Small intestinal tumor outcomes in Apc^1638N^ offspring of fathers consuming different quantities of B vitamins.

		DEF	CTRL	SUPP	ANOVA p
Number of mice with tumors (% incidence)	Female	7 of 15 (46.7)	11 of 22 (50)	9 of 22 (40.9)	0.83
	Male	14 of 17 (82.4)	11 of 14 (78.6)	14 of 17 (82.4)	0.95
	All	21 of 32 (65.6)	22 of 36 (61.1)	23 of 39 (59)	0.85
Number of tumors/mouse*	Female	0.6 ± 0.19	0.59 ± 0.14	0.45 ± 0.13	0.73
	Male	1.35 ± 0.25	1.21 ± 0.24	1.35 ± 0.21	0.9
	All	1.0 ± 0.17	0.9 ± 0.14	0.85 ± 0.14	0.91
Number of tumors that were invasive carcinomas (%)	Female	0 of 9 (0)	0 of 13 (0)	0 of 10 (0)	N/A
	Male	2 of 23 (8.7)	0 of 17 (0)	2 of 23 (8.7)	0.45
	All	2 of 32 (6.3)	0 of 30 (0)	2 of 33 (6.1)	0.38
Average total tumor volume per mouse (mm^3^)*^‡^	Female	12.6 ± 4.3	16.9 ± 4.9	25.1 ± 9.6	0.48
	Male	78.7 ± 28.2	74.9 ± 22.5	70.0 ± 26.4	0.97
	All	47.7 ± 16.0	38.5 ± 9.9	44.7 ± 13.0	0.88
Average volume of tumors (mm^3^)* ψ	Female	21.1 ± 4.5	28.7 ± 3.7	55.3 ± 15.7†	*0*.*04*
	Male	58.2 ± 13.5	57.3 ± 15.6	51.7 ± 16.0	0.94
	All	47.7 ± 10.2	44.9 ± 9.3	52.8 ± 12.0	0.87
Tumor location (% proximal, middle, distal)	Female	88.9, 11.1, 0	76.9, 23.1, 0	80.0, 20.0, 0	0.86
	Male	65.2, 26.1, 8.7	76.5, 17.7, 5.9	77.3, 22.7, 0.0	0.7
	All	71.9, 21.9, 6.3	76.7, 20.0, 3.3	78.1, 21.9, 0.0	0.85

*Values are mean ± SEM.

^‡^ Includes mice with and without tumors (mice without tumors have tumor volume of zero).

ψ Includes only mice with tumors.

† Post–test p = 0.04 vs. DEF and 0.1 vs. CTRL. P_trend_ for paternal diet = 0.04. Paternal diets: DEF, B vitamin deficient; CTRL, B vitamin replete; SUPP, B vitamin supplemented. n = 32–39/gp.

### Weanling offspring hepatic gene expression

Compared to CTRL offspring, DEF and SUPP offspring had 118 (S7 Table) and 133 (S8 Table) differentially expressed hepatic genes respectively (q<0.05). Pathway analysis of these changes revealed a significant enrichment of genes associated with ‘Cardiovascular Disease’, ‘Cell Growth and Survival’ and ‘Lipid Metabolism’ in the DEF comparison. For the SUPP comparison there was a significant enrichment of genes associated with ‘Cancer’, ‘Lipid Metabolism’ and Cellular Development’ (S9 Table). Thirty seven genes were changed in both comparisons.

### Adult offspring hepatic lipid content

**Fig 3:**
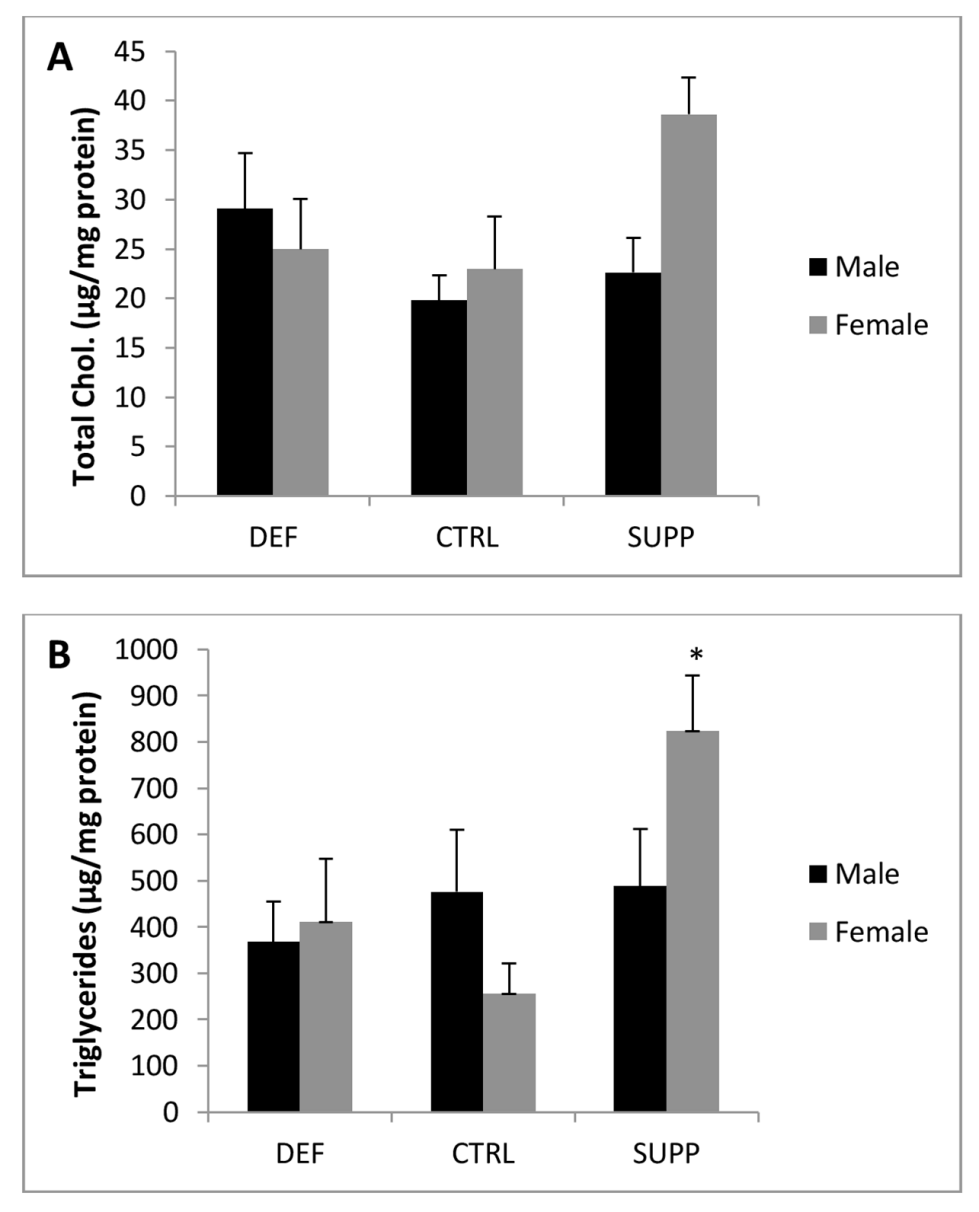
Hepatic lipid concentrations of adult Apc^1638N^ offspring of fathers fed diets differing in B vitamin content. **A)** Hepatic total cholesterol concentration. **B)** Hepatic triglyceride concentration. Paternal diets: DEF, B vitamin deficient; CTRL, B vitamin replete; SUPP, B vitamin supplemented. * denotes significant difference compared to CTRL of same sex (p<0.05). n = 13 per diet group.

In order to understand the functional ramifications of the enrichment of *Lipid Metabolism*-related genes amongst those altered by paternal DEF and SUPP diets (37 of 118 [30%] and 34 of 133 [25%] genes respectively), we measured the hepatic lipid content of adult offspring. Pooling male and female offspring yielded no significant differences in hepatic total cholesterol among offspring of different paternal diet groups (p>0.05). However, when we stratified by sex, female offspring of SUPP fathers tended to have higher total cholesterol concentrations than CTRL (38.6 ± 3.7 vs. 23.0 ± 5.3 vs. μg/mg protein, p = 0.08) (Fig 3A). Hepatic triglycerides (TG) were significantly elevated in offspring of SUPP compared to CTRL-fed fathers (643.7 ± 95.3 vs. 374.5 ± 81.4 μg/mg protein, respectively; p = 0.047). After stratifying according to sex, it was apparent that female offspring were responsible for this effect. Although hepatic TG were not changed amongst male offspring of the different paternal groups (p = 0.997), female offspring of SUPP fathers had a ~3.2-fold elevated TG content compared to CTRL offspring (824.6 ± 118.8 vs. 256.0 ± 64.7 μg/mg protein, respectively; p = 0.009; Fig 3B). The elevation in hepatic lipids seen in female offspring of SUPP fathers was confirmed by Oil Red-O staining, although to a slightly lesser extent (26.4 vs. 13.1% area stained in SUPP and CTRL offspring respectively. P = 0.048. S2 Fig). Hepatic TG and Oil Red-O measures were significantly correlated (R = 0.53, P = 0.04). The elevation in hepatic TG was not accompanied by an elevation in hepatic Tnf, Il1b, IL6 or Ifn (P>0.05. Data not shown).

### Sex-specific hepatic gene expression in offspring

**Fig 4:**
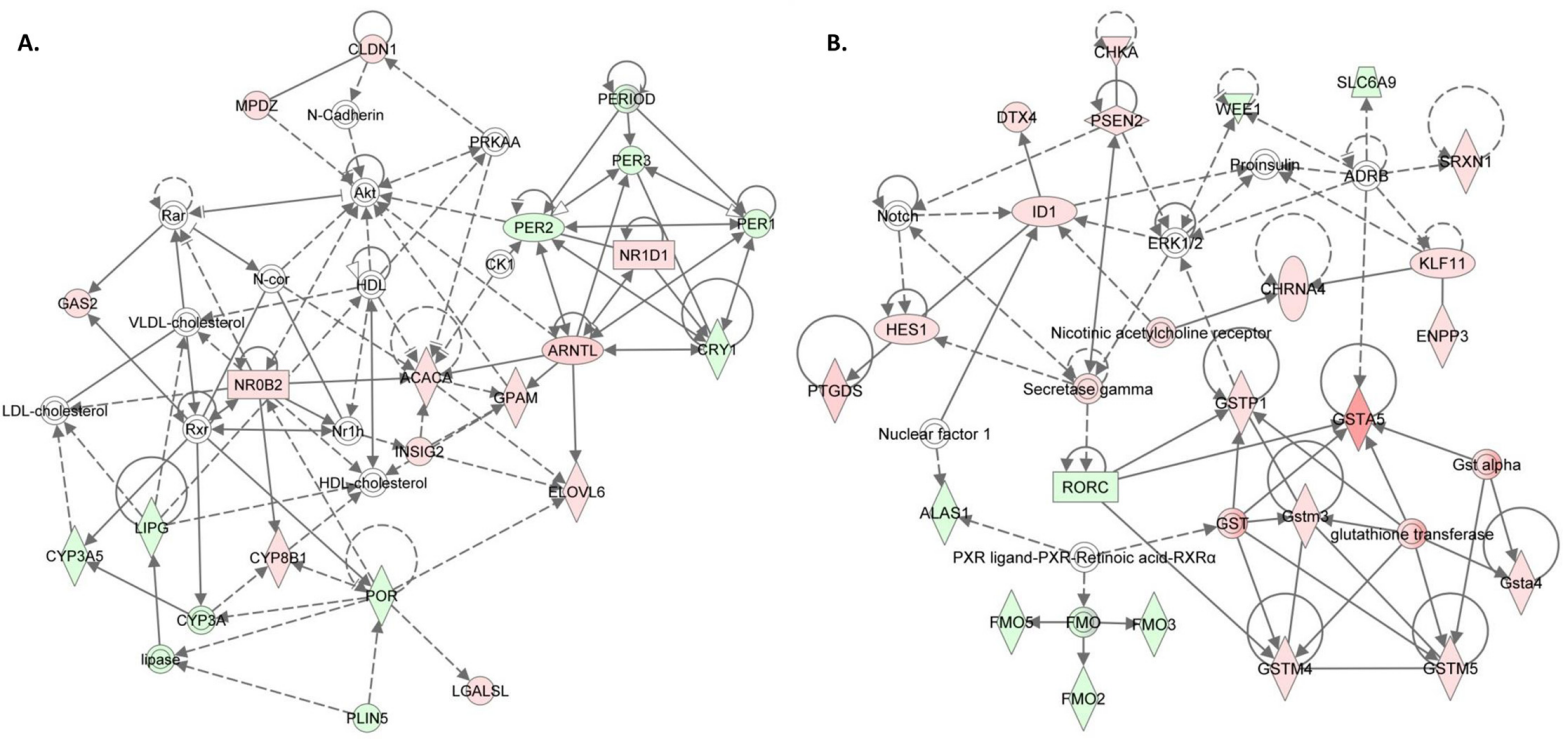
Top networks of differentially expressed genes between CTRL and SUPP offspring unique to females. **A)**. Network associated with ‘Lipid Metabolism’, ‘Behavior’ and ‘Nervous System Development and Function’. Score = 36. **B)**. Network associated with ‘Glutathione Depletion in Liver’, ‘Drug Metabolism’ and ‘Protein Synthesis’. Score = 44. Red = up-regulated in SUPP, Green = down-regulated in SUPP. Unshaded genes are present in the biological network but were not significantly altered in our dataset. The network score is a negative log p value of the Fisher exact test, which is testing whether these genes are grouped by chance.

Because of the apparent sex-specificity of paternal diet’s effect on body weight and hepatic lipids, we reanalyzed our gene expression data after separating by sex. Focusing on the CTRL vs. SUPP comparison for which we observed striking differences in hepatic TG in the elder siblings, there were 219 and 169 genes altered in female and male offspring, respectively. Forty-two of these genes were overlapping, thus 175 were unique to females (S10 Table) and 127 to males. IPA analysis of the 175 female-unique genes again highlighted an enrichment of genes associated with ‘*Lipid Metabolism*’ (52 genes. p = 2.97x10^-8^–8.1x10^-3^ for 63 functions), but also of genes associated with cancer (149 genes. p = 2.05x10^-6^–8.1x10-^3^ for 55 sub categories). Amongst the top Toxicity functions was hepatic steatosis (p = 6.99x10^-4^) with associated genes including *Acaca*, *Cbs*, *Ccnd1*, *Cyp4a11*, *Ddc*, *Gstp1*, *Insig2*, *Por and Rorc*. One of the most robust networks (score = 36) was also associated with the function of ‘*Lipid Metabolism*’ (Fig 4A). This network features several fatty acid-metabolizing genes including *Gpam*, glycerol-3-phosphate acyltransferase, mitochondrial; *Acaca*, acetyl-Coenzyme A carboxylase alpha; *Elovl6*, elongation of long chain fatty acids; *Lipg*, lipase- endothelial; and *Plin5*, perlipin5. Thirteen other genes with roles in lipid, cholesterol and triglyceride homeostasis are also found within this network (S11 Table). Interestingly, the network is also populated with 6 clock genes including *Per1*, period circadian clock 1; *Per2*, period circadian clock 2, *Per3*, period circadian clock 3; *Cry1*, cryptochrome1; *Arntl*, aryl hydrocarbon receptor nuclear translocator-like and *Nr1d1*, nuclear receptor subfamily 1, group D, member 1. The highest scoring network was associated with ‘Glutathione depletion in the liver’ and was populated with 6 glutathione S-transferase encoding genes (Fig 4B). *Acaca*, *ELovl6* and *Gpam* are of particular interest because of their sequential role in *de novo* lipogenesis. In an attempt to understand why these genes were differentially expressed we measured the methylation of select CpG sites in their promoter regions however were unable to detect differences due to group or sexes that could explain the expression differences (S3 Fig).

### Comparison of fathers’ sperm methylation and offspring gene expression

**Fig 5:**
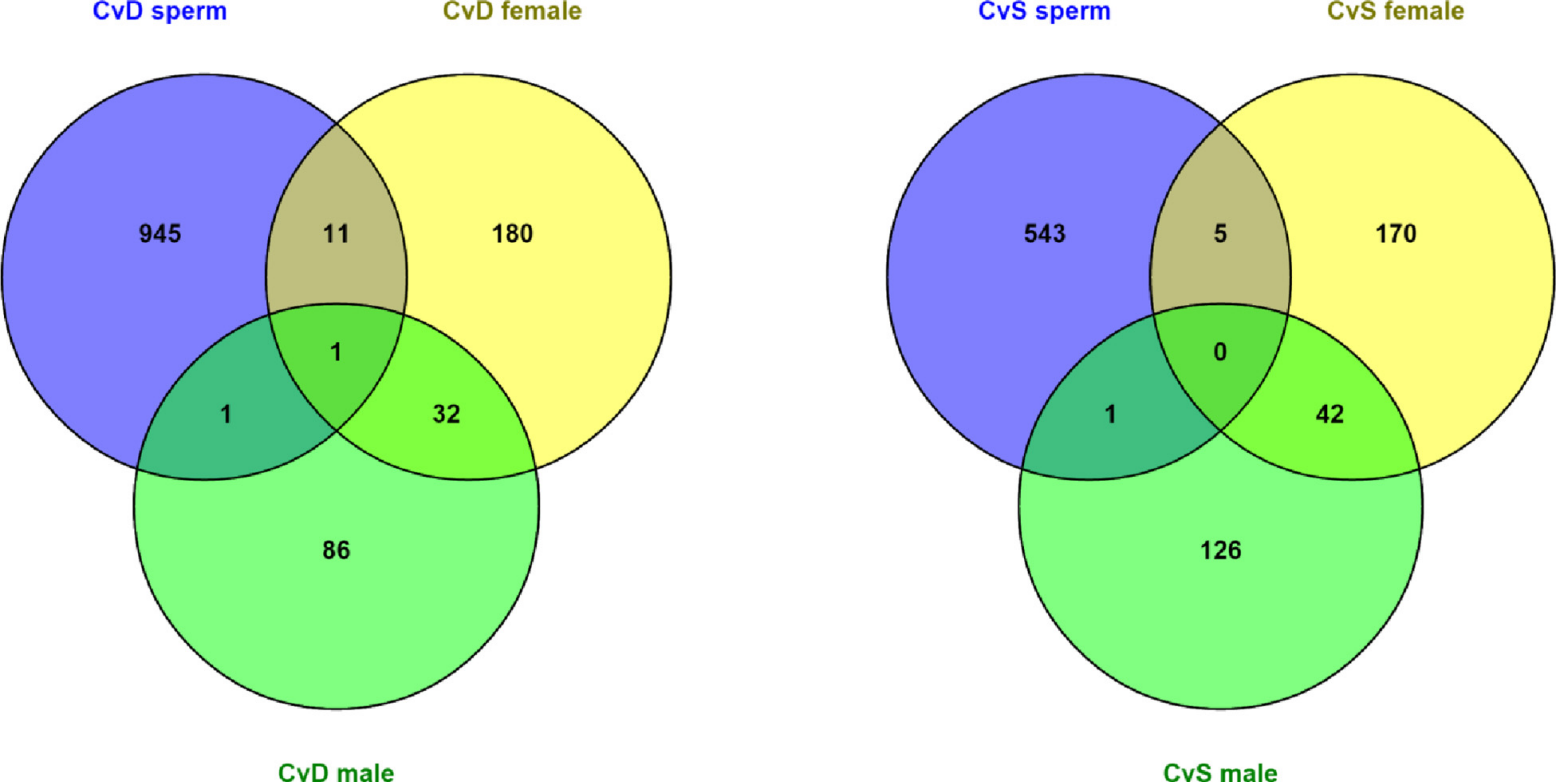
Overlap between differentially methylated genes in sperm and differentially expressed genes in offspring. **A)** CTRL v DEF comparison. Common in sperm and female offspring: Smc4, Spon2, Gpd2, Aurka, Tspan8, Tbc1d10a, Lss, Abhd2, Fdps, Hsd17b7, Dsg1c. Common in sperm and male offspring: Lrrc16a. Common in sperm and female and male offspring: Apol9b. **B)** CTR v SUPP comparison. Common in sperm and female offspring: 1300002K09Rik, Cyp3a16, Ethe1, Igf2, 1810011O10Rik. Common in sperm and male offspring: Col27a1.

To gain an understanding of whether paternal sperm methylation profiles are transmitted to offspring we assessed the commonalities between differentially methylated genes in paternal sperm and differentially expressed genes in offspring. For the DEF vs. CTRL comparison, three genes were both differentially methylated in fathers’ sperm and differentially expressed in offspring liver: *Apol9b*, *Dsg1c*, and *Tspan8*. For the SUPP vs. CTRL comparison, five genes were found to be both differentially methylated in fathers’ sperm and differentially expressed in offspring liver according to the Cufflinks analysis: *1300002K09Rik*, *Cyp3a16*, *Ethe1*, *Igf2*, and *Tspan8*. Considering male and female offspring separately did not increase the overlap between paternal and offspring gene lists for either diet comparison (Fig 5). Of note *Igf2*, a known imprinted gene, was hypermethylated in the sperm of SUPP compared to CTRL fed fathers and downregulated in female offspring of SUPP compared to CTRL fathers.

## Discussion

We previously demonstrated that maternal peri-conceptional supplementation with vitamins B_2_, B_6_, B_12_ and folate significantly attenuates tumor formation in offspring [42]. Based on this finding, and also on accumulating data that altering paternal macro [8, 9] and micronutrient (folate) [10, 11] intake can alter various phenotypes in offspring, we sought to determine whether paternal consumption of vitamins B_2_, B_6_, B_12_ and folate could influence tumor formation in offspring. Contrary to our expectations, paternal consumption of diets containing deficient or supplemental quantities of these vitamins did not alter the likelihood of intestinal tumors forming in their Apc^1638N^ offspring. Although tumor incidence was not affected by paternal diet, the size of tumors did differ between groups in a sex-specific fashion. Specifically, we noted a step-wise increase in tumor volume with increasing paternal B vitamin intake in female but not male offspring. The significance of this finding is unclear but suggests that paternal 1-carbon nutrient intake alters growth of existing neoplasms in female offspring rather than the initiation of new tumors.

Interestingly, we also observed robust sex-specific differences in the body weight of offspring that persisted throughout life; female offspring of fathers consuming DEF and SUPP were 8–10% lighter than those of CTRL fed fathers. In an effort to understand the underlying mechanism for this growth differential, and to investigate the impact of paternal diet on offspring physiology and metabolism in general, we profiled the hepatic transcriptome of weanling offspring. Functional analysis of the genes differentially expressed genes in DEF and SUPP offspring revealed a significant enrichment of genes involved in lipid metabolism. We thus measured the lipid content of offspring liver and found that female, but not male, offspring of SUPP fathers displayed a ~3-fold elevation of hepatic triglycerides (TG) and a trend for elevated cholesterol as well. Importantly, these elevations were observed despite all offspring being maintained on AIN-93G diet which provides only 16% of energy from fat, thus are likely to be exaggerated upon consumption a high fat diet. To put these changes into perspective, others have shown that 15 weeks on a 45% high fat diet increases hepatic triglycerides by only 2-fold in the same strain of mice [43]. This finding is of importance because elevated hepatic triglycerides are associated with insulin resistance [44] and elevated risk of cardiovascular disease [45].

**Fig 6:**
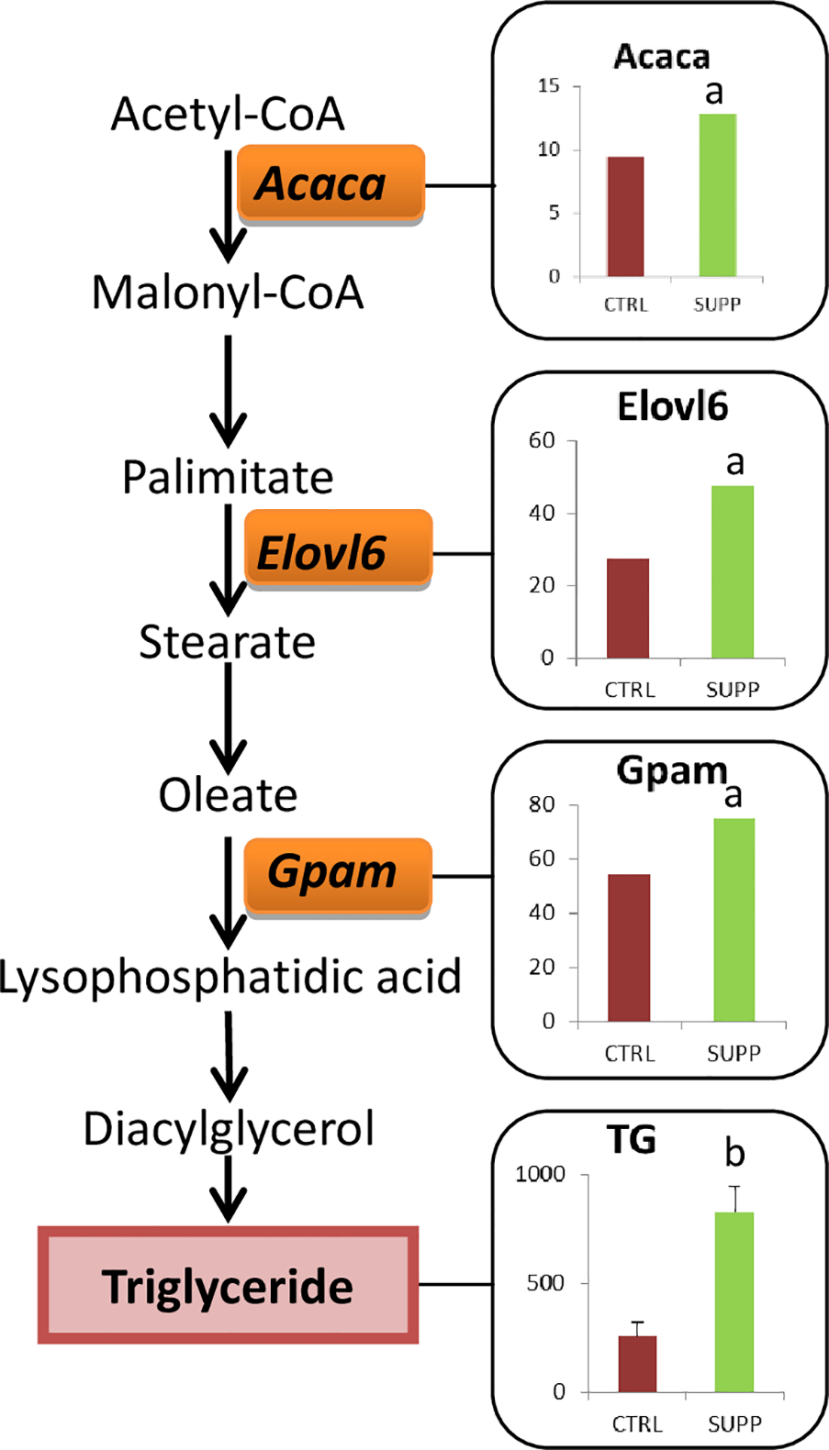
Effect of paternal B vitamin supplementation on hepatic gene expression and triglyceride concentration in female offspring. Gene expression levels are expressed as Fragments Per Kilobase Of Exon Per Million Fragments Mapped (FPKM). Triglycerides are expressed as μg/mg protein. CTRL, control; SUPP, supplemented (father’s diet).^a^ q<0.05, ^b^ p = 0.002 vs CTRL.

To investigate possible mechanisms for the female-specific changes in hepatic TG we reviewed the 175 genes that were changed only in female offspring in response to paternal SUPP diet consumption. Again, there was striking enrichment of lipid metabolizing genes (52 of 175 altered genes), and network analysis highlighted three genes specifically involved in hepatic triglyceride synthesis; *Acaca*, *Elovl6* and *Gpam* (Figs 4 and 6). Of these, *Gpam* is of particular interest because it catalyzes the rate limiting step in TG synthesis and others have shown its expression to be sensitive to maternal diet. Specifically, consumption maternal of a high fat, high sucrose diet induced *Gpam* promoter hypomethylation in pups that was associated with elevated expression of the gene and elevated hepatic TG [46]. Further, an increase in *Elovl6* expression is also likely to elevate hepatic TG as its overexpression has been shown to increase hepatic TG and also hepatic inflammation in mice [47]. Moreover, based on data showing that liver specific knockout of *Acaca* reduces *de novo* fatty acid synthesis and hepatic TG in mice [48], we predict that increased *Acaca* expression could also have contributed to the observed increase in TG in our study. Thus, based on their demonstrated ability to alter hepatic TG, we suggest that the concurrent up-regulation of *Gpam*, *Elovl6* and *Acaca* is a highly plausible mechanism to explain the observed elevation of hepatic triglycerides in female offspring of SUPP fathers. Surprisingly, bisulfite pyrosequencing of regions within the promoter of these genes failed to identify differences due to paternal diet or offspring sex (S3 Fig). Nevertheless methylation of sequences not targeted cannot be ruled out as a cause for the observed differences in gene expression. Alternatively, other epigenetic marks such as histone modifications or the abundance of transcription factors for these genes could be involved.

Our observations cannot establish whether the concurrent changes in lipid metabolism in female offspring are mechanistically linked to the increased tumor volume observed in these animals, but it is certainly feasible. Many important enzymes in lipid metabolism that are upregulated in adipogenesis are also observed to be increased in various types of cancer cells [49] and, in many cases, forced downregulation of their expression leads to cancer cell apoptosis [50, 51].

Also amongst the genes differentially expressed between female offspring of CTRL and SUPP fathers, *Igf2 -*an imprinted gene expressed only from the paternal allele [52]- was down regulated in SUPP offspring. Based on the growth attenuation previously documented in *Igf2* mutant [53] mice, it is plausible that the reduced *Igf2* expression seen here is involved in the reduced body weight observed in these female SUPP offspring. *Igf2* was one of very few genes that were differentially expressed in offspring and differentially methylated in father’s sperm; *Igf2-*associated sequences were relatively hypermethylated in the sperm of SUPP compared to CTRL sires. Thus, it is possible that transmission of this altered methylation state underlies the reduced *Igf2* expression (and body weight) seen in female offspring of SUPP fathers. We profiled the methylation of the H19 DMR, which is downstream of *Igf2* but known to affect its expression, but did not observe any differences in methylation between group or sex. Again, we cannot rule out the possibility that altered methylation of sequences not targeted here could have contributed to the altered expression seen between groups.

Apart from a few genes, including *Igf2*, there was a striking lack of overlap between paternal sperm methylation and offspring gene expression. Similarly others have shown that, despite severe paternal folate deficiency inducing gross structural birth defects, there was very little overlap in sperm methylation and offspring expression when employing similar methods [11]. These findings are consistent with the current dogma that the majority of the male genome is actively demethylated during early embryogenesis [12, 54]. Despite this extensive erasure of methyl marks during development our data suggest that some diet-sensitive changes are not erased and may persist in the developing embryo to alter gene expression. Certainly the application of higher resolution techniques such as bisulfite next-gen sequencing will bring greater clarity to the field.

DNA methylation is certainly not the only epigenetic mark that may carry information from one generation to another. For example, histone methylation was altered in sperm in response to paternal folate deficiency [11] and evidence suggests that histone modifications at developmentally-important loci in sperm can be transmitted to the next generation [55]. There is also evidence that RNA molecules in sperm can affect offspring phenotype [56, 57], thus, these other forms of transgenerational epigenetic inheritance may also be at play in our study. It is also possible that heritable mutations in paternal sperm DNA could impact offspring physiology—although this would likely occur in a random fashion.

The sex specific effects of paternal B vitamin modulation that we observed are consistent with observations of others. For example, paternal high-fat diet induced an impairment of glucose tolerance in only female rat offspring [8]. Although others have shown paternal macronutrient to impact offspring metabolism, this is the first report that altering paternal micronutrients can affect offspring metabolism. Moreover, while others have demonstrated that paternal micronutrients can influence various endpoints in offspring, both studies terminated offspring before birth, thus this is the first demonstration that such alteration in paternal micronutrient can have persistent effects in living offspring that reach over 6 months of age.

Unexpectedly, we found that the two dietary extremes each led to male sires that were 5–10% lower in weight than the males on the control diet even though the animals were pair-fed. The mechanisms underlying this growth differential are unclear but could involve the role of these vitamins as cofactors in energy metabolism. We cannot exclude the possibility that these differences in paternal weight between dietary groups are at least partially responsible for the changes in body weight, lipid metabolism and tumor volume observed in their offspring. However, given the very modest differences in weight, lack of difference in plasma glucose and insulin, the bimodal nature of the effect on paternal weight, and absence of an effect on sperm motility and mating success, this seems an unlikely possibility.

In the current study we utilized diets designed to induce a mild B vitamin depletion diet and moderate level of supplementation four times the basal requirement for mice. Although there is a lot to learn from deficiency studies, their relevance to public health in industrialized countries is fading; in NHANES the prevalence of reproductive age males consuming below the estimated average requirement is only 4–6% [58]. In contrast, because of the high prevalence of foods that are fortified and widespread use of vitamin supplements (35% of US adults use vitamins containing folic acid), the concern is now folate over-nutrition. The average daily intake in the same group is 774–938 μg dietary folate equivalents (DFE), or around double the recommended daily intake of 400 μg DFE. Around 25% of these males consume even more than 800 μg DFE /day [58].

In summary, we have shown that modest alterations in paternal B vitamin intake cause extensive alterations in the methylome of sperm. We have also shown that, within the context of our model, modulation of paternal B vitamin intake prior to mating can influence tumor volume but not incidence or multiplicity in female offspring. Moreover, female offspring of B vitamin supplemented fathers also display elevated hepatic triglycerides despite lower body weight, suggesting diverse impacts on the offspring physiology. Concurrent with these changes we also identified many differentially expressed genes in offspring, several of which likely underlie the elevated TG observed in female offspring. Together with a small but growing body of literature, our data indicate that paternal diet, and in particular one-carbon nutrient intake, can affect various aspects of offspring health. Our observations have raised many questions and clearly much work is needed to characterize fully how paternal diet affects offspring health but such an understanding may allow us tailor paternal nutrition to optimize offspring health and reduce the risk of disease.

## Supporting Information

S1 Fig Body weight of male mice consuming diets with deficient, replete or supplemental quantities of vitamins B_2_, B_6_, B_12_ and folate. After 8 weeks on diet males were cohoused with females to facilitate mating overnight. Food was withheld overnight but provided when males and females were separated during the day. Paternal diets: DEF, B vitamin deficient; CTRL, B vitamin replete; SUPP, B vitamin supplemented. * indicates significant difference compared to CTRL group (p < .05). n = 24-27/gp
(DOCX)

S2 Fig Effect of paternal B vitamin supplementation on hepatic lipids in female offspring. **A)** Average % area covered by Oil Red-O staining for each group measured by ImageJ (n = 5/gp with 3 images per mouse). * Denotes significant difference compared to CTRL. Mean [Range] = 14.6 ± 6.0 [1.9–25.0%], 13.1 ± 5.8 [0.8–22.9%] and 26.4 ± 4.5 [21.3–39.8%] for DEF, CTRL and SUPP offspring respectively. **B, C, D)** Photomicrographs of Oil Red-O stained liver sections from DEF (22.7%), CTRL (22.3%) and SUPP (37.6%) offspring respectively. Images are from mice with the *highest* staining area in each group. Images taken at 100x and scale bar is 200 μm. Paternal diets: DEF, B vitamin deficient; CTRL, B vitamin replete; SUPP, B vitamin supplemented.
(DOCX)

S3 Fig DNA methylation of select genes in offspring liver according to paternal diet. DNA methylation was measured in target genes using bisulfite pyrosequencing. **A)** CpGs 1–14 are located at -359, -357, -347, -337, -334, -320, -314, -304, -296, -288, -280, -274, -267 and -264 bp from the start of *Acaca1* exon 1 respectively. **B)** CpGs 1–9 are located at +36, +59, +50, +27, +14, +10, -3, -14, -40 and -44 bp from the start of *Elovl6* exon 1 respectively. **C)** CpGs 1–19 are located -122, -113, -96, - 89, -77, -73, -64, -61, -52, -50, -48, -45, -41, -39, -33, -31, -28, -26 and -23 bp from start of *Gpam* exon 1 respectively.**D)** CpGs 1–8 are located -3775, -3766, -3754, -3706, -3702, -3699, -3685, -3678 and -3665 bp from start of H19 exon 1 respectively. Unless noted, for each CpG site, 2-Way ANOVA p (group) >0.05 and p (sex) >0.05. ‘a’ denotes p <0.05 for group effect and ‘b’ denotes p v0.05 for sex effect. Unless noted, repeated measures ANOVA for group (considering males and females separately) effect yielded a p>0.05. Paternal diets: DEF, B vitamin deficient; CTRL, B vitamin replete; SUPP, B vitamin supplemented; M male; F female. n = 4 males and 8 females per group.
(DOCX)

S1 Table Pyrosequencing primers. B denotes biotin modification of primer at 5’ end of FWD or 3’ end of RVS primer.
(DOCX)

S2 Table Plasma glucose and insulin concentrations in fathers consuming different quantities of B vitamins. Values are mean ± SEM. n = 10, 13 and 9 respectively.
(DOCX)

S3 Table Regions of the sperm genome differentially methylated in response to B vitamin deficiency. DMR ID is an arbitrary identifier for each differentially methylated region; CTRL, control diet; DEF, B vitamin deficient diet; m1, mean log2(636/532) for CTRL sperm; m2, mean log2(636/532) for DEF sperm; chr:position identifies the location of the DMR within the chromosome using mouse genome build mm9 coordinates; diff, mean log2(635/532) difference across the DMR; qval, q-value—an adjusted p-value indicating the false discovery rate (FDR) calculated by the Wilcoxon rank sum-test. For genes with a qval < 0.1, less than 10% of genes are expected to be false positives; Gene symbol identifies the gene annotated to a DMR and its proximity in bases to the left (-) or right (+) of the DMR up to a distance of 1000 kb where all genome coordinates and gene symbols are derived from mouse genome build mm9. n = 8/gp.
(XLSX)

S4 Table Regions of the sperm genome differentially methylated in response to B vitamin supplementation. DMR ID is an arbitrary identifier for each differentially methylated region; CTRL, control diet; SUPP, B vitamin supplemented diet; m1, mean log2(636/532) for CTRL sperm; m2, mean log2(636/532) for SUPP sperm; chr:position identifies the location of the DMR within the chromosome using mouse genome build mm9 coordinates; diff, mean log2(635/532) difference across the DMR; qval, q-value—an adjusted p-value indicating the false discovery rate (FDR) calculated by the Wilcoxon rank sum-test. For genes with a qval < 0.1, less than 10% of genes are expected to be false positives; Gene symbol identifies the gene annotated to a DMR and its proximity in bases to the left (-) or right (+) of the DMR up to a distance of 1000 kb where all genome coordinates and gene symbols are derived from mouse genome build mm9. n = 8/gp.
(XLSX)

S5 Table Effect of paternal B vitamin intake on measures of reproductive health. * Values are mean ± SEM. Note that number weaned offspring is less than sired offspring due to maternal cannibalism that is common amongst first-time mothers. DEF, B vitamin deficient; CTRL, B vitamin replete; SUPP, B vitamin supplemented.
(DOCX)

S6 Table Plasma insulin and leptin of adult Apc^1638N^ offspring of fathers fed diets differing in B vitamin content. Values are mean ± SEM. Paternal diets, DEF, B vitamin deficient; CTRL, B vitamin replete; SUPP, B vitamin supplemented. Sample size is in parentheses.
(DOCX)

S7 Table Hepatic genes differentially expressed between weanling offspring of fathers consuming control and deficient quantities of B vitamins. Significant differences according to Cuffdiff analysis combining male and female offspring (q<0.05). Expression values are Fragments Per Kilobase Of Exon Per Million Fragments Mapped (FPKM). CTRL, control; DEF, deficient (paternal diet). n = 10/gp.
(XLSX)

S8 Table Hepatic genes differentially expressed between weanling offspring of fathers consuming control and supplemental quantities of B vitamins. Significant differences according to Cuffdiff analysis combining male and female offspring (q<0.05). Expression values are Fragments Per Kilobase Of Exon Per Million Fragments Mapped (FPKM). CTRL, control; SUPP, supplemented (paternal diet). n = 10/gp.
(XLSX)

S9 Table Top functional categories enriched amongst the genes differentially expressed in offspring liver in response to paternal diet. Categories identified by Ingenuity Pathway Analysis®. DEF, B vitamin deficient; CTRL, B vitamin replete; SUPP, B vitamin supplemented (paternal diet). P values represent the range of p values of the sub-categories of each category.
(DOCX)

S10 Table Genes differentially expressed only in female offspring of fathers consuming control and B vitamin supplemented diets. n = 5/gp.
(XLSX)

S11 Table Genes with roles in cholesterol, lipid or triglyceride homeostasis that are amongst those altered in female liver in response to paternal B vitamin supplementation. (DOCX)
