# Correction: Paternal B Vitamin Intake Is a Determinant of Growth, Hepatic Lipid Metabolism and Intestinal Tumor Volume in Female Apc^1638N^ Mouse Offspring

**DOI:** 10.1371/journal.pone.0154979

**Published:** 2016-04-28

**Authors:** Julia A. Sabet, Lara K. Park, Lakshmanan K. Iyer, Albert K. Tai, Gar Yee Koh, Anna C. Pfalzer, Laurence D. Parnell, Joel B. Mason, Zhenhua Liu, Alexander J. Byun, Jimmy W. Crott

The following information is missing from the Funding section: Funding was provided by NCI (National Cancer Institute) 1 R03CA162505-1 with an Administrative Supplement from the Office of Dietary Supplements.
