# Supplementary figures and images for: Paternal B Vitamin Intake Is a Determinant of Growth, Hepatic Lipid Metabolism and Intestinal Tumor Volume in Female Apc1638N Mouse Offspring
Source: PLoS One. 2016 Mar 11;11(3):e0151579. doi: 10.1371/journal.pone.0151579 (PMC4788446; doi:10.1371/journal.pone.0151579)

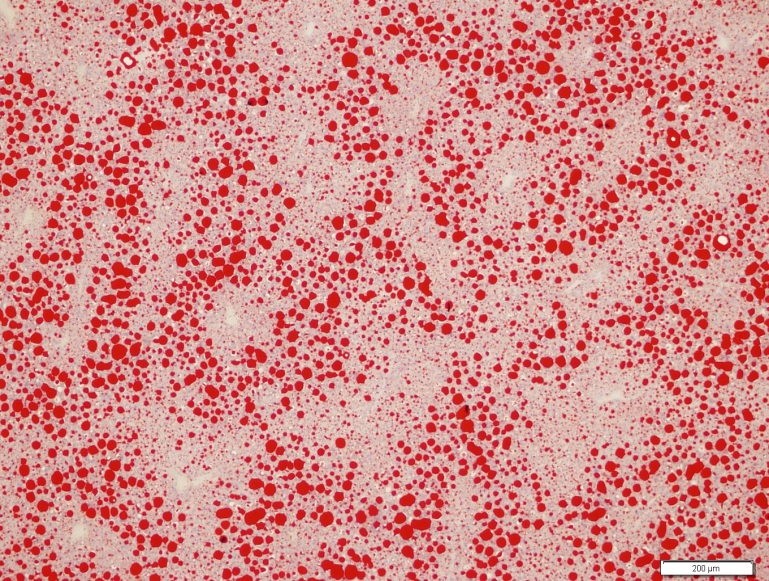


*****

**B**


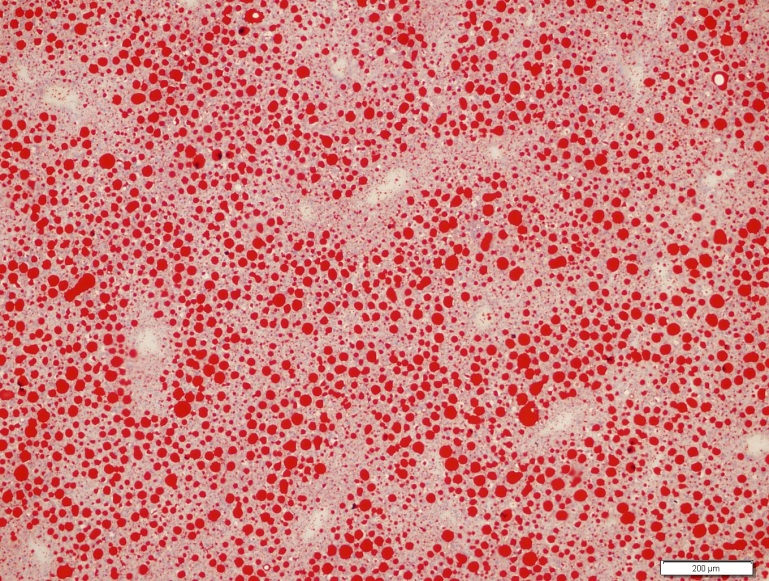

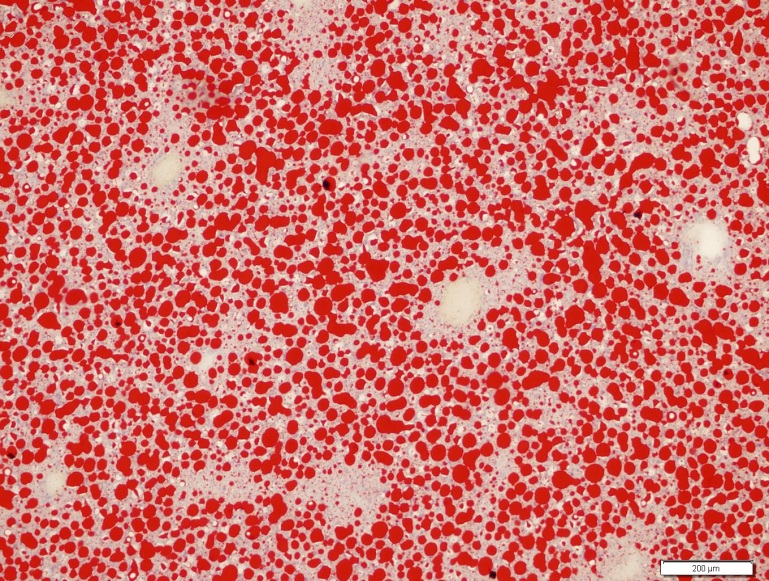


**C**

**D**

Supplement: S2 Fig — A) Average % area covered by Oil Red-O staining for each group measured by ImageJ (n = 5/gp with 3 images per mouse). * Denotes significant difference compared to CTRL. Mean [Range] = 14.6 ± 6.0 [1.9–25.0%], 13.1 ± 5.8 [0.8–22.9%] and 26.4 ± 4.5 [21.3–39.8%] for DEF, CTRL and SUPP offspring respectively. B, C, D) Photomicrographs of Oil Red-O stained liver sections from DEF (22.7%), CTRL (22.3%) and SUPP (37.6%) offspring respectively. Images are from mice with the highest staining area in each group. Images taken at 100x and scale bar is 200 μm. Paternal diets: DEF, B vitamin deficient; CTRL, B vitamin replete; SUPP, B vitamin supplemented. (DOCX) [file pone.0151579.s002.docx]

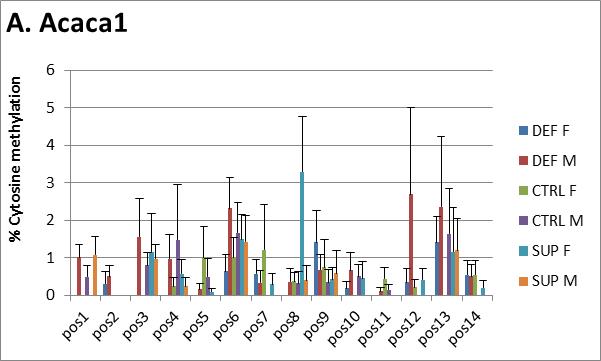

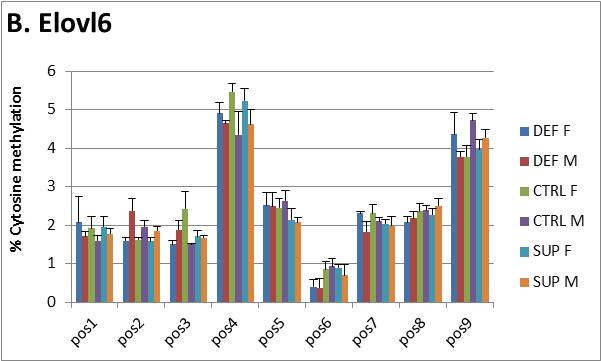


a

b


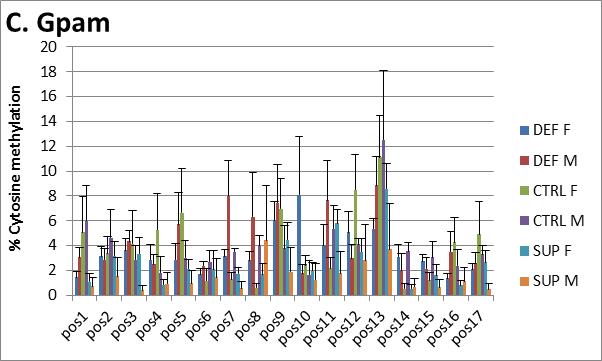

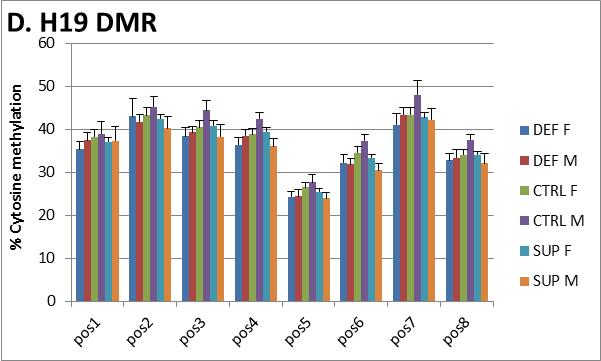


b

a,b

Supplement: S3 Fig — DNA methylation was measured in target genes using bisulfite pyrosequencing. A) CpGs 1–14 are located at -359, -357, -347, -337, -334, -320, -314, -304, -296, -288, -280, -274, -267 and -264 bp from the start of Acaca1 exon 1 respectively. B) CpGs 1–9 are located at +36, +59, +50, +27, +14, +10, -3, -14, -40 and -44 bp from the start of Elovl6 exon 1 respectively. C) CpGs 1–19 are located -122, -113, -96, - 89, -77, -73, -64, -61, -52, -50, -48, -45, -41, -39, -33, -31, -28, -26 and -23 bp from start of Gpam exon 1 respectively.D) CpGs 1–8 are located -3775, -3766, -3754, -3706, -3702, -3699, -3685, -3678 and -3665 bp from start of H19 exon 1 respectively. Unless noted, for each CpG site, 2-Way ANOVA p (group) >0.05 and p (sex) >0.05. ‘a’ denotes p <0.05 for group effect and ‘b’ denotes p v0.05 for sex effect. Unless noted, repeated measures ANOVA for group (considering males and females separately) effect yielded a p>0.05. Paternal diets: DEF, B vitamin deficient; CTRL, B vitamin replete; SUPP, B vitamin supplemented; M male; F female. n = 4 males and 8 females per group. (DOCX) [file pone.0151579.s003.docx]
