# Supplementary material for: Paternal B Vitamin Intake Is a Determinant of Growth, Hepatic Lipid Metabolism and Intestinal Tumor Volume in Female Apc1638N Mouse Offspring
Source: PLoS One. 2016 Mar 11;11(3):e0151579. doi: 10.1371/journal.pone.0151579 (PMC4788446; doi:10.1371/journal.pone.0151579)
Supplement: S1 Table — B denotes biotin modification of primer at 5’ end of FWD or 3’ end of RVS primer. (DOCX) [file pone.0151579.s004.docx]

## Table S1. Primers for pyrosequencing assays.

|  | FWD primer | RVS primer | Sequencing primer |
| --- | --- | --- | --- |
| Acaca1 | GGTTTTGGGTAGGTTATTGTAGT | AATACCACCCTCCCCATCCC_B | GGGTTTAGATAGTAAAATAGTGT (F) |
| Gpam | GTAAGAAGTATGTAGTTTGGGTAGTGTAG | ATATCAACCCTACTAATACTATCACT_B | GATATTAGGTTTATTTTTTATGTTT (F) |
| Elovl6 | B_TAGGTAGGGAGGTTTAAGAGG | ACTCCCAACTCCTAACTCA | CCTAACTCAAAAACTCTC (R) |
| H19DMR | GGGGTTATAAATGTTATTAGGGGGGTAG | AACCCCAAAACCCTATAAATCAAAT_B | TAGGGGGGTAGGATA (F) |

B denotes biotin modification of primer at 5’ end of FWD or 3’ end of RVS primer.
