# Supplementary material for: Paternal B Vitamin Intake Is a Determinant of Growth, Hepatic Lipid Metabolism and Intestinal Tumor Volume in Female Apc1638N Mouse Offspring
Source: PLoS One. 2016 Mar 11;11(3):e0151579. doi: 10.1371/journal.pone.0151579 (PMC4788446; doi:10.1371/journal.pone.0151579)
Supplement: S2 Table — Values are mean ± SEM. n = 10, 13 and 9 respectively. (DOCX) [file pone.0151579.s005.docx]

## Table S2. Plasma glucose and insulin concentrations in fathers consuming different quantities of B vitamins.

|  | **DEF** | **CTRL** | **SUPP** | **p value** |
| --- | --- | --- | --- | --- |
| Insulin (ng/ml) | 3.65 ± 0.95 | 2.45 ± 0.37 | 3.40 ± 0.72 | 0.37 |
| Glucose (μM) | 8.65 ± 0.58 | 9.17 ± 0.43 | 9.63 ± 0.29 | 0.37 |

Values are mean ± SEM. N= 10,13 and 9 respectively.
