# Supplementary material for: Paternal B Vitamin Intake Is a Determinant of Growth, Hepatic Lipid Metabolism and Intestinal Tumor Volume in Female Apc1638N Mouse Offspring
Source: PLoS One. 2016 Mar 11;11(3):e0151579. doi: 10.1371/journal.pone.0151579 (PMC4788446; doi:10.1371/journal.pone.0151579)
Supplement: S5 Table — * Values are mean ± SEM. Note that number weaned offspring is less than sired offspring due to maternal cannibalism that is common amongst first-time mothers. DEF, B vitamin deficient; CTRL, B vitamin replete; SUPP, B vitamin supplemented. (DOCX) [file pone.0151579.s008.docx]

## Table S5. Effect of paternal B vitamin intake on measures of reproductive health

|  | **Diet Group** | | |  |
| --- | --- | --- | --- | --- |
| **Endpoint** | **DEF** | **CTRL** | **SUPP** | **p value** |
| Number of males mated | 25 | 25 | 23 |  |
| Sperm motility (%)* | 26.3 ± 3.3 | 24.0 ± 3.4 | 22.4 ± 3.2 | 0.87 |
| % of males that sired offspring | 52 | 64 | 60.9 | 0.70 |
| % of males with weaned offspring | 44 | 56 | 43.5 | 0.67 |
| No. weaned pups/litter* | 6.2 ± 0.7 | 5.9 ± 0.4 | 7.3 ± 0.6 | 0.21 |
| % Apc^1638N^ pups | 48.5 | 45.8 | 53.4 | 0.63 |
| % Female pups | 52.9 | 51.8 | 52.1 | 1.00 |

* Values are mean ± SEM. Note that number weaned offspring is less than sired offspring due to maternal cannibalism that is common amongst first-time mothers. DEF, B vitamin deficient; CTRL, B vitamin replete; SUPP, B vitamin supplemented.
