# Supplementary material for: Paternal B Vitamin Intake Is a Determinant of Growth, Hepatic Lipid Metabolism and Intestinal Tumor Volume in Female Apc1638N Mouse Offspring
Source: PLoS One. 2016 Mar 11;11(3):e0151579. doi: 10.1371/journal.pone.0151579 (PMC4788446; doi:10.1371/journal.pone.0151579)
Supplement: S6 Table — Values are mean ± SEM. Paternal diets, DEF, B vitamin deficient; CTRL, B vitamin replete; SUPP, B vitamin supplemented. Sample size is in parentheses. (DOCX) [file pone.0151579.s009.docx]

## Table S6. Plasma insulin and leptin of **adult** Apc^1638N^ offspring of fathers fed diets differing in B vitamin content.

|  |  | **Paternal diet** | | |  |
| --- | --- | --- | --- | --- | --- |
|  | Sex | **DEF** | **CTRL** | **SUPP** | p value |
| Insulin (ng/ml) | Female  Male  All | 2.2 ± 0.6 (6)  2.9 ± 0.8 (6)  2.6 ± 0.5 (12) | 2.4 ± 0.9 (7)  1.7 ± 0.6 (5)  2.1 ± 0.6 (12) | 2.9 ± 0.7 (7)  3.3 ± 0.9 (7)  3.1 ± 0.6 (14) | 0.8  0.4  0.4 |
| Leptin (ng/ml) | Female  Male  All | 27.5 ± 5.2 (5)  37.6 ± 5.3 (5)  32.6 ± 3.9 (10) | 37.7 ± 3.0 (6)  31.6 ± 5.9 (7)  34.4 ± 3.4 (13) | 38.4 ± 3.0 (7)  31.6 ± 4.8 (7)  35.0 ± 2.9 (14) | 0.1  0.7  0.9 |

Values are mean ± SEM. Paternal diets, DEF, B vitamin deficient; CTRL, B vitamin replete; SUPP, B vitamin supplemented. Sample size is in parentheses.
