# Supplementary material for: Paternal B Vitamin Intake Is a Determinant of Growth, Hepatic Lipid Metabolism and Intestinal Tumor Volume in Female Apc1638N Mouse Offspring
Source: PLoS One. 2016 Mar 11;11(3):e0151579. doi: 10.1371/journal.pone.0151579 (PMC4788446; doi:10.1371/journal.pone.0151579)
Supplement: S9 Table — Categories identified by Ingenuity Pathway Analysis®. DEF, B vitamin deficient; CTRL, B vitamin replete; SUPP, B vitamin supplemented (paternal diet). P values represent the range of p values of the sub-categories of each category. (DOCX) [file pone.0151579.s012.docx]

## Table S9. Top functional categories enriched amongst the genes differentially expressed in offspring liver in response to paternal diet .

**A. CTRL vs DEF**

| **Diseases and Disorders** | **p value** | **# Molecules** |
| --- | --- | --- |
| Infectious Disease | 8.16E-09 - 1.89E-03 | 20 |
| Cardiovascular Disease | 1.20E-06 - 2.23E-03 | 27 |
| Organismal Injury and Abnormalities | 1.20E-06 - 3.10E-03 | 48 |
| Skeletal and Muscular Disorders | 1.20E-06 - 8.15E-04 | 24 |
| Dermatological Diseases and Conditions | 1.72E-06 - 4.80E-04 | 17 |
| **Molecular and Cellular Functions** | **p value** | **# Molecules** |
| Cellular Function and Maintenance | 2.03E-09 - 2.98E-03 | 42 |
| Cellular Movement | 3.21E-09 - 3.06E-03 | 41 |
| Cell Death and Survival | 4.50E-09 - 3.21E-03 | 53 |
| Lipid Metabolism | 3.98E-07 - 2.84E-03 | 37 |
| Small Molecule Biochemistry | 3.98E-07 - 2.98E-03 | 47 |

**B. CTRL vs SUPP**

| **Diseases and Disorders** | **p value** | **# Molecules** |
| --- | --- | --- |
| Organismal Injury and Abnormalities | 5.64E-04 - 1.24E-02 | 35 |
| Renal and Urological Disease | 5.64E-04 - 1.24E-02 | 5 |
| Inflammatory Response | 5.86E-04 - 1.24E-02 | 19 |
| Cancer | 6.17E-04 - 1.24E-02 | 30 |
| Hematological Disease | 7.64E-04 - 8.64E-03 | 20 |
| **Molecular and Cellular Functions** | **p value** | **# Molecules** |
| Lipid Metabolism | 2.95E-06 - 1.24E-02 | 34 |
| Small Molecule Biochemistry | 2.95E-06 - 1.24E-02 | 42 |
| Cell Cycle | 3.71E-05 - 1.24E-02 | 14 |
| Cellular Development | 4.39E-05 - 1.24E-02 | 29 |
| Cellular Growth and Proliferation | 4.39E-05 - 1.24E-02 | 21 |

Categories identified by Ingenuity Pathway Analysis®. DEF, B vitamin deficient; CTRL, B vitamin replete; SUPP, B vitamin supplemented (paternal diet). P values represent the range of p values of the sub-categories of each category.
