# Supplementary material for: Paternal B Vitamin Intake Is a Determinant of Growth, Hepatic Lipid Metabolism and Intestinal Tumor Volume in Female Apc1638N Mouse Offspring
Source: PLoS One. 2016 Mar 11;11(3):e0151579. doi: 10.1371/journal.pone.0151579 (PMC4788446; doi:10.1371/journal.pone.0151579)
Supplement: S11 Table — (DOCX) [file pone.0151579.s014.docx]

## Table S11. Genes with roles in cholesterol, lipid or triglyceride homeostasis that are amongst those altered in female liver in response to paternal B vitamin supplementation.

| **Gene IDs** | **Evidence** |
| --- | --- |
| Acss2, Rnf144a, Wee1 | Respond to plasma cholesterol lowering in mouse aorta [[1](#_ENREF_1)] |
| Arntl | Sirt6 interacts with Clock:Arntl to control circadian chromatin recruitment of Srebf1, leading to cyclic regulation of fatty acid and cholesterol metabolism genes [[2](#_ENREF_2)] |
| Cbs | Implicated in arteriosclerosis [[3](#_ENREF_3)] |
| Cyp8b1, Nr0b2 | NR0B2 is a negative regulator of CYP7A1 and CYP8B1 [[4](#_ENREF_4)] |
| Insig2 | Cooperative interactions in endoplasmic reticulum between Insigs, Scap and cholesterol allow rapid adjustment by Srebf2 to control rates of cholesterol synthesis and uptake in response to slight deviations from physiological set-point levels, thereby ensuring cholesterol homeostasis [[5](#_ENREF_5)] |
| Lipg | LIPG rs2000813 associates with elevated LDL-C and total cholesterol [[6](#_ENREF_6)] |
| Nampt | Serum visfatin levels from postmenopausal women positively correlated with dietary cholesterol [[7](#_ENREF_7)] |
| Nr1d1 | Directs with Hdac3 a circadian rhythm of histone acetylation and gene expression required for normal hepatic lipid homeostasis [[8](#_ENREF_8)] |
| Per2 | Regulates Pparg and controls lipid metabolism independent of circadian function [[9](#_ENREF_9)] |
| Vcam1 | Proatherogenic postprandial triglyceride-rich lipoproteins increased Vcam1 expression in aortic endothelial cells [[10](#_ENREF_10)] |

**References for Table S10.**

1. Bjorkegren JL, Hagg S, Talukdar HA, Foroughi Asl H, Jain RK, et al. (2014) Plasma cholesterol-induced lesion networks activated before regression of early, mature, and advanced atherosclerosis. PLoS Genet 10: e1004201.

2. Masri S, Rigor P, Cervantes M, Ceglia N, Sebastian C, et al. (2014) Partitioning circadian transcription by SIRT6 leads to segregated control of cellular metabolism. Cell 158: 659-672.

3. Zhang Y, De S, Garner JR, Smith K, Wang SA, et al. (2010) Systematic analysis, comparison, and integration of disease based human genetic association data and mouse genetic phenotypic information. BMC Med Genomics 3: 1.

4. Kim DH, Lee JW (2011) Tumor suppressor p53 regulates bile acid homeostasis via small heterodimer partner. Proc Natl Acad Sci U S A 108: 12266-12270.

5. Sokolov A, Radhakrishnan A (2010) Accessibility of cholesterol in endoplasmic reticulum membranes and activation of SREBP-2 switch abruptly at a common cholesterol threshold. J Biol Chem 285: 29480-29490.

6. Razzaghi H, Tempczyk-Russell A, Haubold K, Santorico SA, Shokati T, et al. (2013) Genetic and structure-function studies of missense mutations in human endothelial lipase. PLoS One 8: e55716.

7. Rahbar AR, Nabipour I (2014) The relationship between dietary lipids and serum visfatin and adiponectin levels in postmenopausal women. Endocr Metab Immune Disord Drug Targets 14: 84-92.

8. Feng D, Liu T, Sun Z, Bugge A, Mullican SE, et al. (2011) A circadian rhythm orchestrated by histone deacetylase 3 controls hepatic lipid metabolism. Science 331: 1315-1319.

9. Grimaldi B, Bellet MM, Katada S, Astarita G, Hirayama J, et al. (2010) PER2 controls lipid metabolism by direct regulation of PPARgamma. Cell Metab 12: 509-520.

10. Sun C, Alkhoury K, Wang YI, Foster GA, Radecke CE, et al. (2012) IRF-1 and miRNA126 modulate VCAM-1 expression in response to a high-fat meal. Circ Res 111: 1054-1064.
