# Supplementary material for: Paternal B Vitamin Intake Is a Determinant of Growth, Hepatic Lipid Metabolism and Intestinal Tumor Volume in Female Apc1638N Mouse Offspring
Source: PLoS One. 2016 Mar 11;11(3):e0151579. doi: 10.1371/journal.pone.0151579 (PMC4788446; doi:10.1371/journal.pone.0151579)
Supplement: Supplementary file 13 [file pone.0151579.s001.docx]

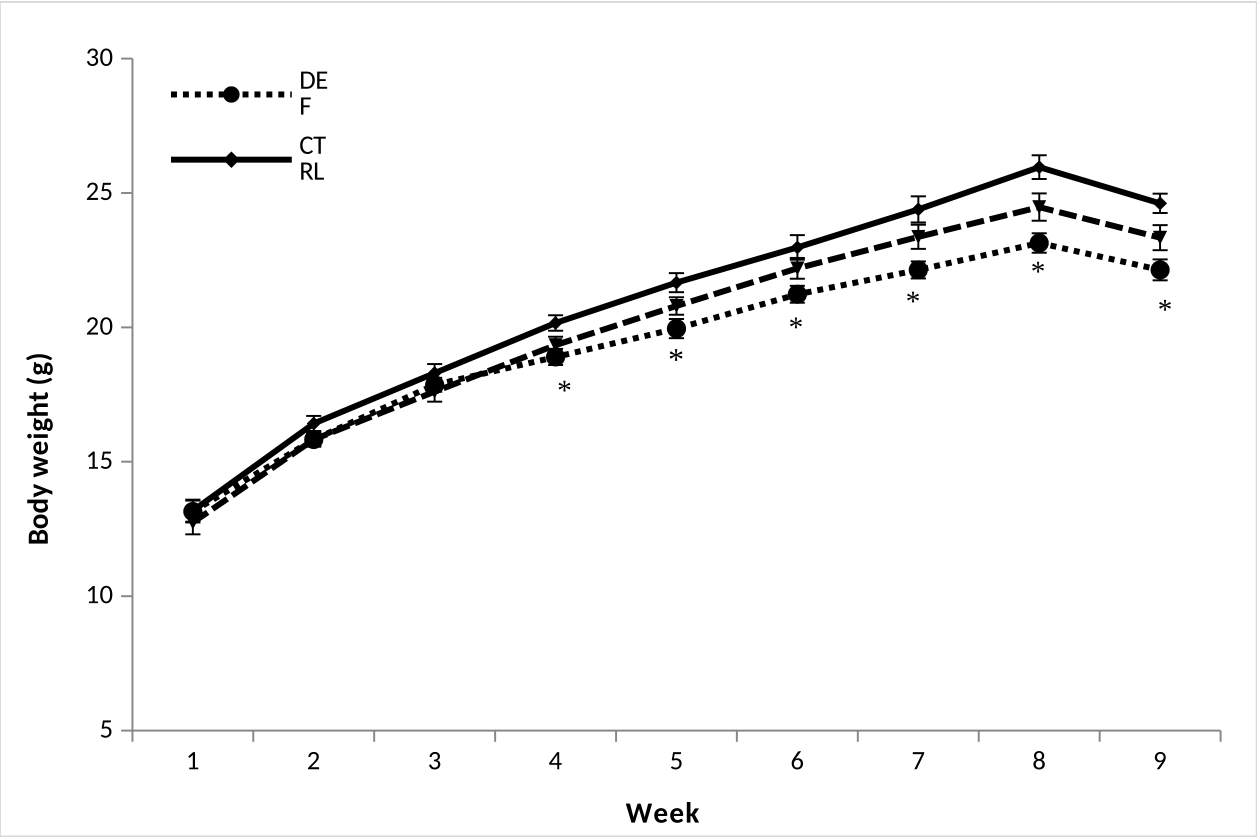

DE
F
CT
RL
30
25
20
15
10
5
Body weight (g)
1
2
3
4
5
6
7
8
9
Week
*
